# New Researches on the Presence of Living Micro-organisms in Normal Muscular Tissues

**Published:** 1919-01

**Authors:** 


**New Researches on the Presence of Living Micro-organisms in Normal Muscular Tissues.** By M. V. GALIPPE. *Bulletin de l'Académie de Médecine de Paris*, July 16, 1918.

Living micro-organisms exist constantly in the cells of all animal and vegetable tissues, and they seem to have quite a preponderant importance in the functional activity of these tissues. It appears therefore quite erroneous to consider sound aseptic tissues as deprived of all parasites.

Microzymas is the name given to these intracellular micro-organisms, and microbiose is the term used in speaking of the general proceedings of their biological activity.

By means of a certain mechanical process (bruising, laceration of the tissues) the author was able to detect some features of this microbiose.

Bruising and laceration of the tissues, as they occur so frequently in war wounds, prove to be sufficient to determine some infection of the wound, without the interference of any external bacilli.

It is ascertained now that living organisms are present in muscular tissues of all animals, that they live there their own autonomous life, and that this may go on even if the tissues happen to be separated from the animal.

Tests were undertaken by the author in order to ascertain this; experiments were carried out on fresh meat brought from slaughter-houses. These are generally in a most dirty state, and meat coming from them is necessarily polluted and contaminated. Furthermore when that meat is loaded on carts or in wagons, the pressure is quite sufficient to enhance the development of all bad effects of the " microbiose ".

Fragments of muscular tissues taken from the inside of a piece of meat were previously passed through a flame to suppress all external infection and then put into culture tubes. After 24 or 48 hrs. numerous strains of streptococci, diplococci, and bacilli, had developed in all the tubes, some very small, some of an ordinary size. The meat had acquired in those tubes a most putrid smell. Among the bacilli could be found certain oval characteristic organisms, whose periphery only could be dyed, and who are considered by the author as a direct result of the microbiose.

Other fragments of meat were crushed intensely before being placed in the culture tubes. In that case microbiose seemed more intense, and the oval bacilli described above were extremely numerous.

Tests were undertaken with frigorific meat and demonstrated that low temperatures do not kill the micro-organisms deposited on the pieces of meat, nor those existing normally in the tissues. As long as the meat is frozen, the vitality of these organisms is considerably slackened, but they revive as soon as the temperature rises.

